# In this issue: Volume 114, Issue 8, August 2023

**DOI:** 10.1111/cas.15908

**Published:** 2023-07-16

## Chromatin structure related to oncogenesis

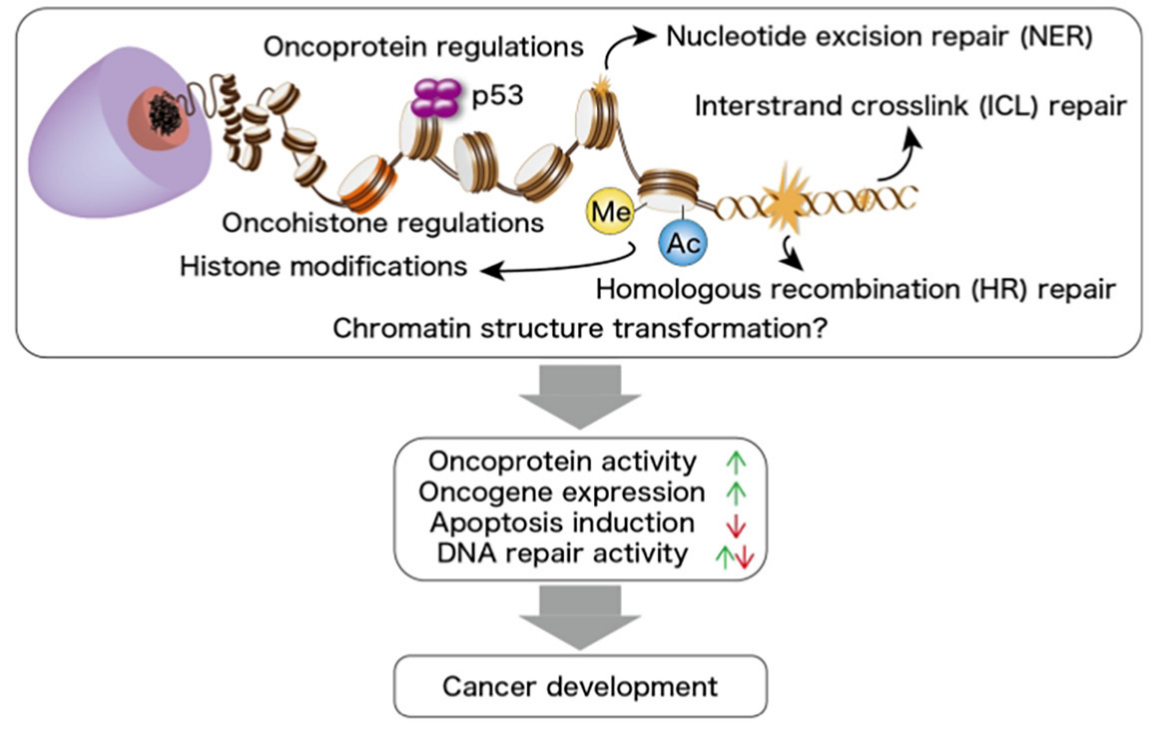

Human DNA is organized in a structure known as chromatin, which plays a vital role in preserving the integrity of the genetic information. This complex arrangement involves DNA being tightly wound around specialized proteins called histones. Changes in the chromatin framework due to mutations, i.e., changes caused by alterations in the gene sequence, can have profound implications on the structure of DNA, leading to disruption of crucial processes, altered gene regulation, and varied interactions with other proteins.

In this review, Matsumoto et al. present a consolidated account of the recent developments in cancer research with a special focus on chromatin proteins and processes that help stabilize the DNA and its structure. They also discuss the different mutations occurring in these proteins, their effects on healthy cells, and the mechanisms underlying the transformation of healthy cells into cancerous cells because of these effects.

The researchers first discuss the role of histones in essential processes like gene regulation, DNA organization, and facilitation of DNA interactions with other proteins. They then explore the different mutations that affect these elements and initiate a cascading set of events that eventually leads to the development of cancer.

Next, the researchers focus on the tumor suppressor protein p53, a molecule that plays a pivotal role in responding to cellular stress. p53 combats cellular stress by activating crucial genes and altering DNA organization, in order to safeguard the integrity of DNA. Mutations in p53 have been associated with the manifestation of different kinds of cancers.

DNA in eukaryotic cells is constantly exposed to various harmful factors and needs repair mechanisms to restore its functionality. Understanding DNA damage and repair mechanisms is therefore crucial, as defects in DNA repair not only promote the development of cancers but also offer resistance against treatments. The researchers therefore discuss these mechanisms and highlight two important DNA repair pathways—the homologous recombination repair pathway, which repairs double‐strand breaks in DNA (fault in this pathway is commonly associated with breast cancer), and the nucleotide excision repair pathway, which repairs damages due to UV light and environmental factors (fault in this pathway is commonly associated with skin cancer).

Although further studies are required to unravel the intricacies of the association between chromatin and cancer development, the insights collated in this review are valuable for the development of newer and more specialized therapies for the treatment of cancer.

https://onlinelibrary.wiley.com/doi/full/10.1111/CAS.15850

## GPAM mediated lysophosphatidic acid synthesis regulates mitochondrial dynamics in acute myeloid leukemia

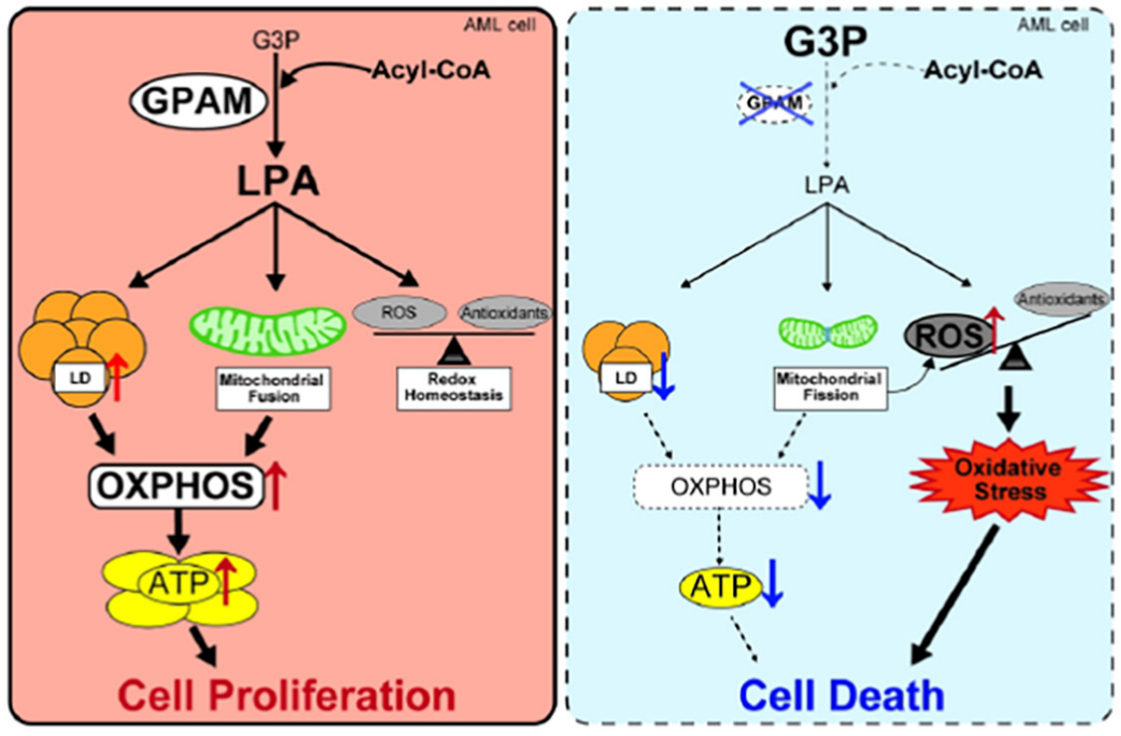

Acute myeloid leukemia (AML) is an aggressive type of blood cancer characterized by the abnormal growth of white blood cells. Unfortunately, AML has a poor prognosis and limited treatment options. However, one promising area of research focuses on the metabolic processes of these cancer cells, particularly their energy production in the mitochondria.

Cancer cells, including those in AML, have distinct metabolic characteristics that set them apart from normal cells. Specifically, the function of mitochondria, the energy‐producing organelles in cells, differs significantly between AML cells and normal cells. This provides a rationale for targeting mitochondrial metabolism in AML treatment. Alterations in how mitochondria divide and fuse have been linked to the development and progression of cancers such as AML, as they can make tumor cells proliferate at higher rates or be more resistant to chemotherapy. However, no AML‐specific molecules regulating these distinct metabolism and mitochondrial dynamics have been identified.

In this study, researchers focused on the role of fat metabolism abnormalities, and how mitochondria use this energy to divide and fuse. In particular, they found that AML cells have heightened activity in synthesizing a lipid called lysophosphatidic acid (LPA), which is mediated by the enzyme glycerol‐3‐phosphate acyltransferases (GPAM).

By targeting GPAM, the scientists were able to disrupt fat metabolism in AML cells, leading to impaired mitochondrial function of cancer cells while leaving healthy cells intact. This suggests that targeting GPAM‐mediated lipid metabolism in AML cells could be an effective therapeutic strategy for this type of cancer.

Current treatment options for AML suffer from limited efficacy, high toxicity, and frequent relapse. Advancing targeted treatments that specifically focus on cancer cell metabolism and energy production holds great promise for the development of more effective and safer therapeutic approaches in treating AML.

https://onlinelibrary.wiley.com/doi/full/10.1111/CAS.15835

## CHMP4A stimulates CD8+ T‐lymphocyte infiltration and inhibits breast tumor growth via the LSD1/IFNβ axis

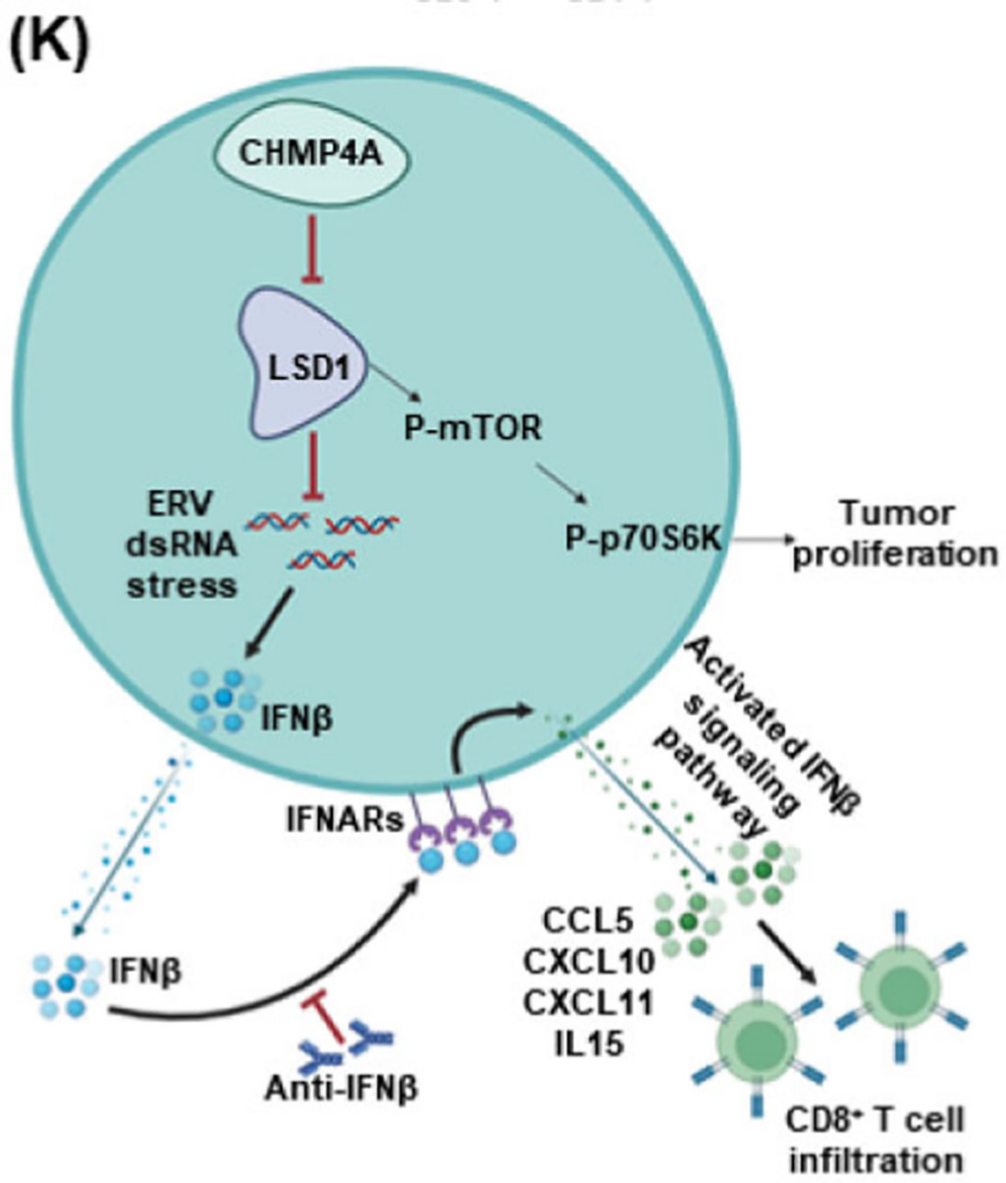

Cancer immunogenicity—a subject of intense medical research in recent years—is the property of cancer cells that determines their likelihood of being detected and eliminated by the immune system. Previous studies have reported that higher level of CD8+ T lymphocytes—a type of tumor‐infiltrating immune cells—are indicative of better clinical outcomes in patients with breast cancer. However, the molecular mechanisms behind this effect seen with CD8+ T lymphocytes in breast cancer remains largely unknown.

In this study, Song et al. applied bioinformatics analyses to identify key genes involved in the CD8+ T‐lymphocyte‐mediated immune response against breast cancer. Their analyses yielded four key genes: *CHMP4A*, *CXCL9*, *GRHL2*, and *RPS29*. But *CHMP4A* that encodes chromatin modifying protein 4A stood out; they found that higher levels of *CHMP4A* expression were associated with longer overall survival in patients with breast cancer.

They conducted further experiments in cell lines and animal models that mimicked breast cancer to understand how *CHMP4A* expression affects the associated CD8+ T‐lymphocyte‐mediated immune response. Their results showed that *CHMP4A* expression slows down the growth of breast cancer cells by recruiting and sending the CD8+ T lymphocytes into the breast cancer.

*CHMP4A* expression was also found to reduce the expression of an enzyme called lysine‐specific demethylase 1; this effect further caused the accumulation of a viral RNA called human endogenous retrovirus (HERV). HERV accumulation, in turn, triggered the production of signaling molecules called interferon‐beta (IFNβ). IFNβ, through multiple intermediary biomolecules, cued the CD8+ T lymphocytes to infiltrate the breast cancer. Through their comprehensive experiments, the authors essentially delineated the molecular mechanisms underlying CD8+ T‐lymphocyte‐mediated immune response against breast cancer.

In summary, these findings highlight the importance of *CHMP4A* in CD8+ T‐cell infiltration and suggest its role as a potential target for improving the effectiveness of immunotherapy against breast cancer. The study also suggests that understanding the interactions among the molecular components responsible for different anti‐tumor effects can help researchers develop new strategies to enhance the body's natural defense mechanisms for fighting breast cancer more effectively.

https://onlinelibrary.wiley.com/doi/full/10.1111/CAS.15844

